# Screening of Microbial Isolates from Tomato Plants (*Solanum lycopersicum* L.) for Bioprotective Potential: From Isolation to Food Model System Application

**DOI:** 10.3390/foods14213713

**Published:** 2025-10-30

**Authors:** Laura Rabasco-Vílchez, Araceli Bolívar, María Julia Ruiz, Narjes Harrazi, Jérôme Mounier, Emmanuel Coton, Luis M. Medina, Fernando Pérez-Rodríguez

**Affiliations:** 1 Department of Food Science and Technology, UIC Zoonosis y Enfermedades Emergentes ENZOEM, ceiA3, Universidad de Córdoba, 14014 Córdoba, Spain; 2 Laboratorio de Inmunoquímica y Biotecnología, Centro de Investigación Veterinaria de Tandil (CIVETAN), CONICET, CICPBA, Facultad de Ciencias Veterinarias, UNICEN-Campus Universitario, Tandil B7000, Argentina; 3 Research Laboratory “Technological Innovation and Food Security-LR22 AGR01”, Higher Institute of Food Industries of Tunisia (ESIAT), University of Carthage, Tunis 1002, Tunisia; 4 INRAE, Laboratoire Universitaire de Biodiversité et Écologie Microbienne, University Brest, F-29280 Plouzane, France

**Keywords:** bio-preservation, post-harvest, shelf-life, isolate, antimicrobial, antifungal, acidic conditions, *Bacillus*, *Pseudomonas*, lactic acid bacteria

## Abstract

This study explores tomato agri-food residues as sources of bacteria with bioprotective potential to enhance product shelf-life and safety. A total of 245 bacterial strains were isolated, comprising predominantly *Pseudomonas* (52%) and *Bacillus* (44%) spp., with lactic acid bacteria (LAB) present at lower levels (4%). The antimicrobial activity of these isolates was assessed against pathogenic and spoilage bacteria and phytopathogenic molds. Notably, the *Bacillus* isolate TRB1-7 exhibited moderate activity against *L. monocytogenes* (inhibition halo diameter: 10.64 mm), while *Pseudomonas* and LAB isolates showed limited or no inhibition. Antifungal assays highlighted significant antifungal potential for *Bacillus* isolates. Results showed that 16% and 15% of the 245 isolates inhibited *F. oxysporum* and *C. acutatum* growth, respectively. Nine of these isolates underwent acid-adaptation and were evaluated against the selected molds using Potato Dextrose Agar (PDA) at pH 4.0 to simulate tomato conditions. Only isolate BRZ3-2, identified as *B. aerius*, was adapted to acidic conditions and inhibited *F. oxysporum* by 25%. Experiments on tomato-based agar at the same pH showed no inhibition by *Bacillus* isolates. These results suggest that tomato microbiota harbors acid-tolerant *Bacillus* strains with potential for post-harvest bio-preservation. Further studies on strains TRB1-7 and BRZ3-2 are required to develop effective bioprotective applications.

## 1. Introduction

Tomato (*Solanum lycopersicum* L.) is one of the most important crops in the European Union (EU) [1], with a per capita consumption that has remained stable over the past decade at approximately 14 kg [2]. Damaged and spoiled tomatoes are also major contributors to food loss and waste, which is recognized as an urgent environmental and social challenge in modern societies, affecting both high-income and low-income communities. Microbial contamination of tomato plants is influenced by multiple factors during pre-harvest and post-harvest stages. Soil and irrigation water quality play critical roles, as contaminated or untreated water, along with soil bearing fecal matter or improperly composted manure, can disseminate foodborne pathogens and fungal spores onto vegetables and fruits [3]. Agricultural practices, including poor sanitation during harvesting and handling, further contribute to the microbial load. Moreover, environmental conditions, particularly temperature, humidity, and physical damage to fruits, facilitate fungal growth and pathogen entry [4].

In the context of a societal demand for reduced pesticide use, bioprotective cultures, which are microorganisms, mainly bacterial species, used to inhibit pathogen growth and mitigate microbiological spoilage in food, represent a natural alternative of interest. The application of bioprotective microorganisms has been widely demonstrated for fermented products, such as dry-cured meats and ripened cheeses, as well as fish products [5,6,7,8,9]. However, research on vegetables and fruits is scarcer [10]. The suitability of bioprotective cultures to control post-harvest microorganisms, reduce spoilage, and/or improve food safety, still requires further scientific development. The selection of the isolates of interest and their capacity to be biologically active under the food product conditions is determinant for their potential application [11]. Moreover, the source of the microorganism can also play a critical role in its performance, as strains isolated from similar matrices or environments may display enhanced adaptability and competitiveness [12].

In vegetable ecosystems, the microbiome plays a paramount role in the defense mechanisms of plants, protecting them against diseases [13]. Different concepts have been proposed in the area of microbial–host interactions, such as “soterobionts”, which have been described as microorganisms that can enhance the host plant’s immune system and protect it against diseases through their modulation capacity in the microbial ecosystem [14]. Applying bioprotective cultures derived from this endogenous microbiota could be an efficient strategy to prevent post-harvest diseases and extend food shelf-life, consequently reducing food waste [15].

An earlier comprehensive review of tomato-associated microbial communities developed in our laboratory [11] highlighted *Bacillus* and *Pseudomonas* spp. as promising bioprotective microorganisms. Isolates of these genera from tomato plants remain under-investigated in comparison to other microbial communities and sources. The latter work also mentioned that the well-known lactic acid bacteria (LAB), commonly used as bioprotective cultures in several foods, are less abundant in these vegetable matrices. In this regard, the isolation of these microorganisms from tomato plants has been previously reported. For instance, Pérez-Rodriguez (2020) [16] isolated different *Pseudomonas* species (*P. fluorescens*, *P. thivervalensis*, and *P. brassicacearum*) from tomato rhizosphere and roots. Other authors have further reported the presence of *Pseudomonas* species on tomato fruits, indicating their persistence beyond the root system [17,18]. In addition, Chaouachi et al. (2021) [19] described the isolation of several *Bacillus* species, including *B. amyloliquefaciens*, *B. vallismortis*, *B. pseudomycoides*, *B. velezensis*, and *B. subtilis*, from both tomato fruits and roots. Regarding LAB, Fessard and Remize (2019) [20] identified strains belonging to *Leuconostoc citreum* and *Lactiplantibacillus plantarum* in tomato fruits, underscoring the microbial diversity associated with this crop.

The screening of candidate bioprotective cultures first requires assessing their antimicrobial capacity against bacterial or fungal targets, generally performed using in vitro antimicrobial assays such as the spot-on-lawn method, first described by Gratia in 1946 and later modified and adapted in several studies [21]. This technique has also been reported in the literature for evaluating antifungal activity [22,23].

The use of artificial food model systems has become increasingly important as an intermediate, food-oriented approach for evaluating microbial behavior, considering the effect of food-related factors. For example, Verheyen et al. (2018, 2020) [24,25] developed fish-based model systems encompassing different microstructures (liquids, emulsions, gelled emulsions) that mimic the compositional, physicochemical, and rheological properties of different ready-to-eat fish products. Possas et al. (2018) [26] developed a simulated meat product to assess the impact of pH, sodium chloride, and sodium nitrite on *L. monocytogenes* under high hydrostatic pressure (HHP) treatments. Garnier et al. (2018) [27] developed a cheese-mimicking system for high-throughput antifungal activity screening. These food-based systems allowed the study of the isolated effects of different compositional and microstructural factors influencing microbial behavior, thus providing quantifiable (metric) variables for the development of accurate predictive models [24]. Moreover, this approach is closer to the reality of the matrix, which is crucial, as it was shown that the effectiveness of an isolate under synthetic conditions cannot be extrapolated to actual matrices [28]. The strategy of employing food-based model systems can be extended to vegetables, offering cost-effective tools for investigating and quantifying microbial behavior in simulated vegetable matrices. However, most studies have focused on enhancing the culture and recovery of endogenous microorganisms from plants by formulating solid and liquid vegetable-based media using tomato, carrot, cabbage, or pumpkin extracts as nutrient sources [29]. Their formulations demonstrated comparable efficacy to conventional media. Similarly, innovative approaches using plant substrates have emerged, such as the use of leaves from sunflowers [30] or from cactus (*Opuntia ficus-indica* (L.) Mill.) and succulent plants (*Aloe vera* (L.) Burm.f. and *Aloe arborescens* Mill.), which efficiently enriched root-associated bacterial populations, underscoring their ecological relevance [31]. Tomato juice agar, a classic vegetable-based medium, has also been evaluated as a sustainable nutrient source for fungal and yeast cultivation [32,33].

The present study aimed to isolate LAB, *Bacillus*, and *Pseudomonas* spp. from different anatomical parts of tomato plants and to systematically screen their antimicrobial activity against foodborne pathogenic bacteria and spoilage molds responsible for tomato losses and waste. The antimicrobial activity was evaluated using various in vitro techniques widely reported in the literature, in combination with tomato-based agar as an experimental food model system. The use of this model, which mimics tomato composition, represents a valuable approach to support the in vitro validation of the antimicrobial activity of the selected microorganisms under conditions that simulate real produce environments.

## 2. Materials and Methods

### 2.1. Plant Material

Healthy long-cycle tomato plants of the ‘Harrison’ and ‘Mistela’ cultivars were collected. Plants were cultivated over five months, from January to May, in Almería, Spain. The sampling was conducted randomly, with the complete tomato plants (including roots, rhizosphere, fruits, leaves, and stems) carefully uprooted. Each plant was collected separately and packaged in sterile plastic bags for shipment to the laboratory. The samples were stored at 5 °C and processed within the following days, before any visible signs of decay appeared.

### 2.2. Bacterial Isolation from Tomato Plant

**Figure 1:**
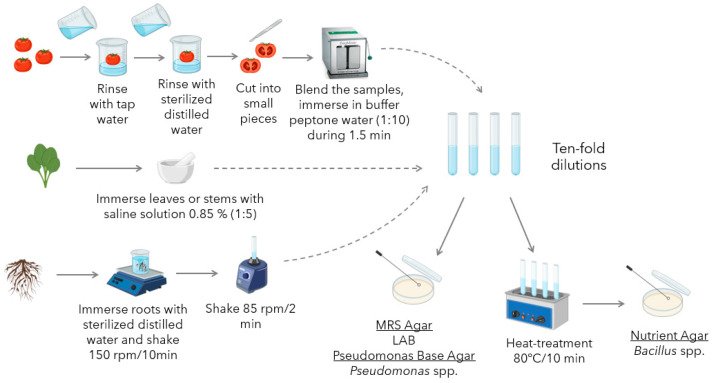
Workflow for the isolation and selective cultivation of microorganisms from tomato fruits, leaves, stems, and roots.

Bacterial isolation from tomato plant microbiota was carried out based on methods reviewed in a previous work [11]. Sample preparation and bacterial isolation protocols (Figure 1) were specific to the anatomical part of the plant to be analyzed, and *Bacillus*, *Pseudomonas* spp., and LAB were selected as the target microbial groups.

#### 2.2.1. Sample Preparation

Isolation of microorganisms from tomato fruit was performed using a modified version of the method described by Abd-Alla et al. (2011) [34]. Fresh tomatoes were thoroughly washed with tap water, followed by distilled water. The fruits were then aseptically cut into small pieces using a sterile scalpel. These pieces were immersed in 0.85% sterilized saline buffer (1:10 dilution) and homogenized for 2 min using a stomacher blender (BagMixer^®^ 400 CC, Interscience, Saint Nom, France). For bacteria isolation from roots and rhizospheres, the procedure followed was mainly focused on the one described by Sunera et al. (2020) [35]. The procedure was modified to enhance soil detachment from the roots and rhizospheres by increasing the agitation speed. A sample of 10 g of roots or rhizosphere was mixed with 90 mL of deionized sterile water and agitated on an incubator shaker (New Brunswick^TM^ Innova^®^ 42, Eppendorf, Hamburg, Germany) for 10 min at 150 rpm. Subsequently, 1 mL of the resulting suspension was transferred to 9 mL of deionized sterile water and further agitated on a vortex for 2 min at 85 rpm.

Finally, to isolate endophytic bacteria from the green tissues of tomato plants, the procedure described by Yang et al. [36] was implemented. To isolate epiphytic bacteria, this procedure was modified by omitting the disinfection step. Leaf or stem samples of approximately 3 to 5 g were collected. The collected tissues were thoroughly rinsed with sterilized distilled water to remove surface contaminants. Subsequently, the samples were homogenized in 0.85% NaCl (Sigma-Aldrich, Darmstadt, Germany) solution using a sterile mortar and pestle.

#### 2.2.2. Bacteria Isolation

After sample preparation, as described in the previous section, a series of ten-fold dilutions (10^−1^ to 10^−4^) from the homogenized samples were prepared using sterile 0.85% saline solution. Aliquots (100 μL) from each dilution, including the undiluted extract, were spread-plated onto the following selective media: de Man, Rogosa, and Sharpe (MRS) agar (Oxoid, Basingstoke, UK) for LAB; *Pseudomonas* Base Agar supplemented with Cetrimide, Fucidin, and Cephalosporin (CFC) (Oxoid, Basingstoke, UK) for *Pseudomonas* spp.; and Nutrient Agar (NA) (Oxoid, Basingstoke, UK) for *Bacillus* spp.

To isolate *Bacillus* spp. and eliminate non-spore-forming microorganisms, the dilutions intended for NA plating were subjected to heat treatment. The samples were placed in a water bath at 80 °C for 10 min, allowing only heat-resistant spores to survive.

The inoculated plates were then incubated at 30 °C for 48 h, with NA and *Pseudomonas* Base Agar under aerobic conditions, and MRS Agar in a 10% CO_2_ atmosphere. Figure 1 presents a graphical summary of the procedure followed for sample preparation and microbial isolation.

### 2.3. Selection and Storage of Isolated Bacterial Colonies

After the incubation period, bacterial colonies were selected based on their distinctive morphological characteristics. The selection process involved a thorough visual inspection, considering colony morphology, pigmentation, and size to identify specific characteristics of the microbial groups under investigation. To maintain the viability and genetic stability of the isolated strains for future studies, the selected colonies were preserved using a standardized cryopreservation method. Each isolate was suspended in sterile 20% (*v*/*v*) glycerol (Sigma-Aldrich, Darmstadt, Germany) with MRS broth (Oxoid, Basingstoke, UK) for LAB, and Nutrient broth (Oxoid, Basingstoke, UK) for *Bacillus* and *Pseudomonas*, then stored at −80 °C.

### 2.4. Antibacterial Activity Assay

#### 2.4.1. Bacterial Inoculum Preparation

The antibacterial activity of the isolated bacteria was evaluated against foodborne pathogenic and spoilage bacteria obtained from the Spanish Type Culture Collection (CECT, Valencia, Spain). The bacterial strains used in this study included the pathogenic species *S. enterica* subsp. *enterica* Typhimurium (CECT 722), *L. monocytogenes* (CECT 4032), and *Escherichia coli* O157:H7 (CECT 8295), as well as the spoilage bacterium *Pectobacterium carotovorum* subsp. *carotovorum* (CECT 225). The pathogenic strains were selected because they represent the main bacterial groups commonly associated with foodborne diseases [37,38,39].

For the preparation of the bacterial inoculum, stock cultures were pre-cultured separately under static conditions for 24 h in Tryptone Soya Broth (TSB) (Oxoid, Basingstoke, UK) for *S. enterica* and *E. coli* O157:H7, and Brain Heart Infusion (BHI) (Oxoid, Basingstoke, UK) for *L. monocytogenes* and *P. carotovorum* at 37 °C. Afterwards, a 24 h-subculture was made for each microorganism under the same incubation conditions, followed by a third subculture incubated for 18–20 h.

#### 2.4.2. Antibacterial Assay

The antagonistic activity assessment method used in this study was the spot on-lawn method, described by Denkova et al. (2017) [40]. Initially, Petri dishes containing selective or differential media were prepared to support the growth of the target microorganisms. Subsequently, three 2 µL drops of the isolated microorganisms from different parts of tomato plant were spotted onto the prepared media and incubated to allow for spot development. Concurrently, soft agar (0.7%) was prepared by combining 6 mL of broth (TSB for *S. enterica* and *E. coli* O157:H7, and BHI for *L. monocytogenes* and *P. carotovorum*) with 0.7% agar, autoclaved, and maintained at approximately 50 °C to prevent solidification. Following spot development, 100 µL of the target bacteria suspension was thoroughly mixed with the prepared soft agar and carefully poured over the Petri dish containing the developed spots. The plates were then incubated at 37 °C for 24 h to enable target microorganism growth and potential formation of inhibition zones around the spots. After the incubation period, the diameters of both the inhibition zones and the spots were measured using a digital caliper (Fisher Scientific, Madrid, Spain; accuracy: ±0.01 mm). In cases for which large inhibition zones overlapped, the procedure was repeated using a single drop placed at the center of the Petri dish. This modification allowed clearer visualization and more precise measurement of the inhibition zone. Positive results were classified according to Del Valle et al. (2019) [41], based on the following differences between the diameter of the inhibition zone surrounding the spot and the diameter of the spot:

- No inhibition (<1 mm).
- Low inhibition (≤10 mm).
- Moderate inhibition (11–21 mm).
- High inhibition (>21 mm).

### 2.5. Antifungal Activity Assay

#### 2.5.1. Fungal Inoculum Preparation

The antifungal activity of the isolated bacteria was studied against *Fusarium oxysporum* and *Colletotrichum acutatum*, two phytopathogenic and spoilage mold species affecting tomatoes. The tested strains were obtained from the Spanish Type Culture Collection (CECT, Valencia, Spain) and corresponded to *F. oxysporum* f. sp. *lycopersici* CECT 2715 and *C. acutatum* CECT 21099.

Initially, the molds were cultivated in Potato Dextrose Broth (PDB) (Oxoid, Basingstoke, UK) for a period of seven days at 28 °C with continuous agitation at 120 rpm (New Brunswick^TM^ Innova^®^ 42, Eppendorf, Hamburg, Germany). After the incubation period, the resulting conidia suspensions were carefully filtered through a sterile filter to remove mycelial fragments. The concentration of conidia in the filtrate was then adjusted to 10^6^ conidia/mL with an optical microscope using a Neubauer counting chamber.

#### 2.5.2. Antifungal Assay

The antifungal activity assay was performed on Potato Dextrose Agar (PDA) (Oxoid, Basingstoke, UK) plates. A 5 µL drop of the adjusted conidia suspension (10^6^ conidia/mL) and a 5 µL drop of the isolated bacterial culture (10^6^ CFU/mL) were carefully spotted 2 cm apart on the surface of the PDA. The inoculated plates were then incubated at 28 °C for five days. Each test was performed in triplicate. Additionally, growth controls for each mold (in triplicate) and each bacterial isolate (in duplicate) were carried out. Following incubation, inhibition was evaluated with the following two parameters: (i) the distance between the drop containing the bacterial culture and the drop containing the spore suspension and (ii) the reduction in fungal colony diameter compared to the control. When no significant distance between bacterial and fungal colonies were observed, only the second parameter was considered. Examples of both criteria are shown in Figure S1.

For the second criterion, the diameter of each colony was measured using a digital caliper. Finally, the percentage of fungal growth inhibition was calculated using the average of the three replicates according to Equation (1):

(1) $$Inhibition\;(\%)=\left(\frac{\;control\;colony\;diameter-\;test\;colony\;diameter}{control\;colony\;diameter}\right)\times 100$$

Inhibition was considered significant when the reduction in fungal colony size was greater than 20% compared to the control, as proposed by Yen et al. (2006) [42].

### 2.6. Adaptation of Bacterial Isolates to Low pH

Nine *Bacillus* isolates showing notable antifungal activity during the antifungal assay were selected for adaptation to low pH. Stock cultures were reactivated by streaking onto NA and incubated for 24 h at 30 °C. Subsequently, the isolates were inoculated into 5 mL of BHI broth and incubated for another 24 h at 30 °C. The pH adaptation process began with inoculating 100 μL of the reactivated cultures into 5 mL of BHI broth adjusted to different pH values using HCl 0.1 M. The tubes were incubated at 30 °C until visible growth (turbidity) was observed. The pH was progressively lowered from the original value (7.4 ± 0.2) in 0.5-unit increments until reaching a final value of 4.0.

The acid-adapted bacterial isolates were then tested for fungal inhibition using the procedure described in Section 2.6. The assays were conducted on PDA at pH 4, which was prepared by adding HCl 0.1 M after sterilization under sterile conditions.

### 2.7. Antifungal Activity Assay on Tomato-Based Agar

The antifungal activity of the nine selected bacteria was also evaluated against *F. oxysporum* and *C. acutatum* on tomato-based agar (TBA) to mimic the compositional and physicochemical conditions found in this product.

The TBA medium was prepared by combining commercial organic tomato juice (pasteurized and free from salt, lemon juice, and additives) (Huerto de Sabor, Navarra, Spain) with an agar solution (40 g/L) in a 1:1 ratio. The antifungal activity was evaluated on TBA at pH 4.0 (the initial pH of the medium) and pH 6.0 (adjusted with NaOH 1 M).

Firstly, inhibition experiments were carried out in 24-well plates. Briefly, 0.2 mL of bacterial inoculum and 2 mL of TBA were added to each well to obtain a final concentration of 10^7^ CFU/mL. Once the medium was solidified, the plates were incubated at 30 °C for 24 h. After incubation, a 5 µL spot of *F. oxysporum* or *C. acutatum* conidial suspension was added to the agar surface, and plates were incubated again at 25 °C until fungal growth was observed (approximately 5–7 days). Antifungal assays were also performed individually in Petri dishes for the isolates showing inhibition in multi-well tests, following the procedure described in Section 2.6. This allowed quantification of the inhibition halo and the distance between the colony and the fungus.

Each inhibition experiment with the nine selected bacteria against *F. oxysporum* and *C. acutatum*, as well as bacteria and mold controls, was performed in triplicate.

### 2.8. Molecular Identification of Selected Bacterial Isolates

Colonies of the selected bacterial isolates were purified on NA (Condalab, Madrid, Spain) to proceed with their molecular identification. The selected bacteria were identified by 16S rRNA gene sequencing with the universal primers 27F (5′AGAGTTTGATCMTGGCTCAG3′) and 1492R (5′CGGTTACCTTGTTACGACTT3′). Genomic DNA of isolates was extracted using the InstaGene Matrix kit (Bio-Rad, Hercules, CA, USA). PCR reactions were performed in a final volume of 25 µL containing 0.2 µL of My Taq DNA polymerase enzyme (Bioline, Meridian Bioscience, Memphis, TN, USA), 5 µL of 5X Buffer, 1 µL of each primer (10 µM), 7.8 µL of molecular water, and 10 µL of template DNA. PCR amplifications were performed with the Eppendorf Mastercycler X50 (Hamburg, Germany). The cycles used are as follows: 5 min at 95 °C; 35 cycles of 30 s at 95 °C, 30 s at 55 °C, and 90 s at 72 °C; and 3 min at 72 °C.

The resulting PCR products were visualized on a 1% (*w*/*v*) agarose gel stained with Midori Green Xtra (Nippon Genetics Europe GmbH, Duren, Germany) for 1 h at a constant voltage of 80 V in 1X AccuGENE TAE buffer (Lonza, Basel, Switzerland). Finally, the PCR products were purified using a purification kit (DNA Clean & Concentrator-5, Zymo Research, Irvine, CA, USA) and sent for sequencing (STAB Vida, Caparica, Portugal). The obtained sequences were aligned using NCBI-BLAST (https://blast.ncbi.nlm.nih.gov/Blast.cgi) (accessed on 15 January 2023). Only sequence similarities above 97% were considered significant for bacterial identification at the species-level.

## 3. Results

### 3.1. Bacteria Isolation and Selection from Tomato Plant

A total of 245 bacterial isolates were obtained from the tomato plant microbiota (Table S1), comprising 52% *Pseudomonas* spp., 44% *Bacillus* spp., and 4% LAB. In terms of anatomical distribution, over half of the isolates were isolated from the green parts of the plant, with 60% from leaves and 13% from stems. Additionally, 12% of the isolates were obtained from tomato fruit, while 15% were recovered from the rhizosphere. Table 1 shows the distribution of the obtained isolates. As shown in Table 1, *Pseudomonas* was the most abundant microbial genus in all anatomical parts, with most isolates coming from the green parts of the plant (leaves and stems).

### 3.2. Antibacterial Activity

The inhibition of pathogenic and spoilage bacteria by the various isolates was evaluated based on the diameter of inhibition halos (mm) surrounding bacterial colonies, which served as indicators of antimicrobial activity. The results were categorized in varying degrees of inhibition according to Del Valle et al. (2019) [37], as mentioned in Section 2. As summarized in Table 2, only 12 bacterial isolates out of 245 demonstrated antimicrobial activity. Among them, low inhibition was observed in 11 isolates, while moderate inhibition was recorded for a single isolate, TRB1-7 (10.64 mm).

When comparing the inhibition zones produced by *Pseudomonas* and *Bacillus* isolates, the latter exhibited inhibition zones (difference between the diameter of the halo and the diameter of the colony) ranging from 5.00 mm to 10.64 mm, whereas *Pseudomonas* isolates showed zones between 4.22 mm and 9.38 mm.

Regarding the influence of the source on antimicrobial activity, TRB1-7—a rhizosphere-derived isolate—was the only one showing moderate inhibition. In contrast, leaf-derived isolates exhibited only low inhibition zones.

More specifically, while some positive results were observed against *L. monocytogenes* and *P. carotovorum*, no inhibition was observed in *S. enterica* and *E. coli*. Against *L. monocytogenes*, inhibition zones ranged from 4.22 mm to 10.64 mm. The *Bacillus* isolate TRB1-7 showed the strongest inhibition (moderate) against *L. monocytogenes* (10.64 mm). For *P. carotovorum*, inhibition zones ranged from 5.00 mm to 8.27 mm, all classified as low inhibition.

These results suggest that *Bacillus* and *Pseudomonas* isolates have some inhibitory effects on both target microorganisms, but their effectiveness remains limited, particularly against *P. carotovorum*. In contrast, LAB was the isolate group that presented the lowest abundance and a reduced inhibitory capacity.

### 3.3. Antifungal Activity

Antifungal inhibition results are presented in Tables S2 and S3 (Supplementary Data). Results are based on both antifungal activity evaluation criteria described in Materials and Methods and illustrated in Figure S1.

Overall, 16% (39) and 15% (38) of the 245 isolates inhibited fungal growth compared to the control for *F. oxysporum* and *C. acutatum*, respectively.

The maximum inhibition against *F. oxysporum* was observed for the isolate BT1-2, reaching 39.63%. Other isolates also showed similar inhibition percentages, such as TFB2-2, TFB3-1, BT1-2, BT2-1, BRZ3-2, and TP1G, all belonging to the *Bacillus* genus. Six *Pseudomonas* showed the capacity to inhibit mold growth; however, only the THP8-6 isolate exhibited a notable inhibition rate above 20% (23.62%). Regarding LAB, two isolates, THA2-1 and THA2-2, both obtained from leaves, demonstrated inhibitory activity against mold growth. However, the reduction levels were relatively moderate, with THA2-1 inhibiting growth by 16% and THA2-2 by 17%.

For *C. acutatum*, isolate THB5-3, belonging to *Bacillus* genus, exhibited the highest inhibitory activity (75.57% fungal inhibition), followed by the TTB4-1 and THB6-3 isolates, both isolated from aerial parts, which resulted in inhibition percentages of 74.52% and 73.94%, respectively. Six *Pseudomonas* isolates also considerably reduced the growth of *C. acutatum*, with isolate THP8-6 showing the highest reduction (48.77%). Finally, regarding LAB, the following two isolates obtained from leaves exhibited high-antifungal activity: isolates THA2-2 and THB6-3 inhibited fungal growth by 58.75% and 34.69%, respectively.

### 3.4. Antifungal Activity at Low pH

Only isolate BRZ3-2 could adapt to pH 4.0. The antifungal activity of the nine selected *Bacillus* isolates was therefore evaluated at pH 6.0. These experiments were only based on measuring the diameter of the fungal colony to determine the percentage of inhibition.

**Figure 2:**
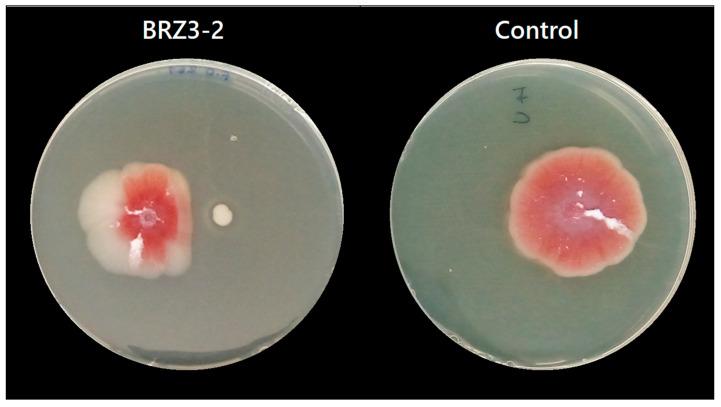
Positive antifungal activity result of the isolate *B. aerius* BRZ3-2 against *F. oxysporum* at pH 4.0.

For *F. oxysporum*, antifungal results were obtained at pH 6.0 and pH 4.0, while for *C. acutatum,* inhibition results were only obtained at pH 6.0. The results showed that at pH 6.0, seven of the nine tested *Bacillus* isolates inhibited the growth of *F. oxysporum* (Figure S2), but at pH 4.0 this activity was almost completely lost, except for isolate BRZ3-2, which developed antifungal activity under both conditions (Figure 2) as it was adapted to pH 4.0. From these results, the isolates TFB2-2, TFB3-1, BT1-2, BT2-1, BRZ3-2, and TP1G stood out, exhibiting inhibition percentages ranging from 38% to 40% at pH 6.0, whereas at pH 4.0, isolate BRZ3-2 showed 25% inhibition (Table 3).

In contrast, all isolates inhibited *C. acutatum* at pH 6.0, with isolate TFB2-2 exhibiting the highest inhibitory activity (41% fungal inhibition), followed by BT1-2, which achieved 35% inhibition. However, at pH 4.0, no inhibition was observed (Table 3). Results of the antifungal activity of the selected *Bacillus* spp. strains at pH 6.0 against *C. acutatum* are presented in Figure S3.

### 3.5. Antifungal Activity on Tomato-Based Agar

The antifungal activity of the selected *Bacillus* spp. against *F. oxysporum* and *C. acutatum* on TBA was first evaluated in 24-well plates. 

The results are presented in Figure S4. Overall, mold growth inhibition was observed at pH 6.0 for the TFB3-1, BT1-2, BT1-4, and BRZ3-2 isolates.

These results were confirmed individually in Petri dishes following the procedure described in Section 2.6. Among these isolates, TFB3-1 achieved the highest inhibition against *F. oxysporum* (38%), followed by BT1-4 and BT1-4 (37% and 34%, respectively). Unlike the results obtained on PDA, on TBA at pH 4.0 the TFB3-1 isolate did not inhibit mold growth. Positive inhibition results on TBA at pH 6.0 are presented in Table 4 and in Figure S5.

The relevant inhibition results against *C. acutatum* are summarized in Table 5. All *Bacillus* isolates exhibited antifungal activity, with seven demonstrating complete inhibition (100%) of fungal growth. In contrast, strain BT1-4 showed a markedly lower inhibitory effect, reducing the diameter of *C. acutatum* by only 23.11%. These findings are also presented in Figure S6.

### 3.6. Molecular Identification of Selected Bacillus spp. Isolates

The nine *Bacillus* spp. isolates showing notable antifungal activity were identified by 16S rRNA gene sequencing. Sequencing of the 16S rRNA region confirmed that these bacteria belonged to the *Bacillus* genus and had similarities with *B. amyloliquefaciens*, *B. velezensis*, and *B. aerius*. All isolates showed almost 100.00% identity with sequences deposited in the GenBank database. Notably, based on the current European Food Safety Authority (EFSA) list of qualified presumption of safety (QPS) biological agents, both *B. amyloliquefaciens* and *B. velezensis* have QPS status, which recognizes these microorganisms as safe for use, with no safety concerns for humans, animals, or the environment. This QPS status highlights the safety of these two *Bacillus* species. Thus, they could be used as bioprotective microorganisms. Nevertheless, as *B. aerius* is not in the QPS list to this date, further studies are required to ensure its safety. Table 3 shows the 16S rRNA gene sequencing results of the nine selected bacteria. The antifungal activity results of these isolated bacteria against *F. oxysporum* and *C. acutatum* on PDA medium without pH adjustment are summarized in Tables S1 and S2, respectively.

## 4. Discussion

A comprehensive bioprotective potential assessment of a total of 245 bacteria isolated from different anatomical parts of healthy tomato plants was carried out in this work. In general, the results showed that *Pseudomonas* spp. and *Bacillus* spp. are relevant microbial groups in the microbiota of tomato plants and that some strains exhibited antimicrobial activity against either pathogenic bacteria or spoilage molds, which supports the idea of a microbiome with the potential to support an active defense of the plant against different deleterious microorganisms. This finding is consistent with the fact that *Pseudomonas spp.* and *Bacillus spp.* are ubiquitous in the rhizosphere and phyllosphere of tomato plants [43]. These bacteria are typically part of the natural plant-associated microbiota and can colonize tissues through root uptake and aerial deposition [44]. The antibacterial activity observed in this study is in line with previous evidence showing that *Bacillus* spp. can inhibit *L. monocytogenes*, although the extent of inhibition varies among strains. Nithya et al. (2012) [45] and Senbagam et al. (2013) [46] reported stronger activity, with inhibition zones between 21 mm (*Bacillus* Ec1) and 35 mm (*B. cereus*), while *B. velezensis* BUU004 exhibited moderate inhibition of around 17 mm. In contrast, our findings, comparable to those of Vadakedath et al. (2019) [47] (7 mm), indicate a lower antibacterial effect, possibly reflecting strain-dependent differences in antimicrobial compound production.

Regarding the antifungal activity, Rocha et al. (2017) isolated various *Bacillus* strains from tomato plants and evaluated their antifungal activity against *F. oxysporum*, obtaining inhibition percentages comparable to those observed in the present study [48]. Similarly, other authors have reported the antifungal potential of *Bacillus* strains isolated from different sources against *F. oxysporum*. Various studies have confirmed the antifungal capabilities of *Bacillus* strains from diverse origins. For example, *Bacillus subtilis* AKPS2 isolated from a resistant banana cultivar exhibited strong antagonism against *F. oxysporum* f. sp. cubense, with an inhibition percentage of 61% [49]. Other *Bacillus* species, including *B. siamensis*, *B. amyloliquefaciens*, and *B. subtilis*, isolated from organic wastes, showed antifungal activities against *F. oxysporum* and *Alternaria alternata*, linked to enzymatic activity and secretion of antimicrobial metabolites [50]. Additionally, Bacillaceae isolates from Cuban wheat not only inhibited *Fusarium* species but also promoted wheat growth through the production of growth-promoting and lytic enzymes [51]. Compared to these results, the 39.63% inhibition achieved in our study demonstrates moderate but meaningful antifungal activity, highlighting the potential of *Bacillus* as an antifungal agent.

Similarly, the antifungal efficacy of *Bacillus* spp. has also been demonstrated against *C. acutatum* in other matrices, including apple orchards [52] and grapevines [53]. Notably, the results from this study, with a maximum inhibition of 75.57%, surpass some field applications such as the 47% reduction reported for *Bacillus subtilis* ACB-69 [54]. The antifungal activity of *Bacillus* spp. is primarily attributed to the production of cyclic lipopeptides such as surfactins, fengycins, and iturins [55]. These compounds have distinct modes of action on fungal cell membranes: surfactins disrupt membrane structure through biosurfactant activity, iturins form ion-conducting pores leading to membrane permeability changes, and fengycins alter membrane organization and permeability [56]. Additionally, *Bacillus* species produce volatile organic compounds (VOCs) that induce oxidative stress in fungi by generating reactive oxygen species, which damage intracellular components and lead to cell death [57]. These VOCs also inhibit fungal spore germination, reduce virulence, and affect enzymes related to cell wall and membrane integrity [58].

In line with our findings, Wang et al. (2020) [59] reported similar inhibition results for a *B. amyloliquefaciens* strain isolated from the surface of tomato fruit when tested against *C. acutatum*. Moreover, the antifungal activity of *Bacillus* spp. against other phytopathogenic molds, such as *R. stolonifer* and *A. flavus*, has also been documented [60,61,62,63,64,65,66].

In relation to plant anatomy, our results seemed to highlight the antimicrobial activity of isolates from the rhizosphere compared to the activity found in bacteria isolated from leaves. In this sense, ref. [67] considered that, overall, the plants had a distinctive and genotype-dependent rhizosphere microbiome with higher abundances of known beneficial bacteria such as *Pseudomonas* and *Rhizobium*. The rhizosphere microbiome plays a significant role in resistance against fungal population such as *Verticillium* or *Macrophomina*. In addition, studies have shown that an imbalance in the rhizosphere microbiome can increase the plant’s susceptibility to foliar diseases. For example, and directly related to our case, dysbiosis of the soil microbiome in tomatoes increased the severity of bacterial spots on leaves, highlighting the importance of the root microbiome in the systemic defense of the plant [68]. The isolate exhibiting the higher activity on PDA was the TRB1-7 strain (*Bacillus* sp.), isolated from the rhizosphere.

On the other hand, although our results showed lower antimicrobial activity for leaf isolates, the existence of this inhibition is consistent with some published cases, such as the mediation of the phyllosphere microbiota in the resistance of the cocoa plant to *Phytophthora* [69].

The microbial species identified in this work have also been reported in association with tomato plants [48], including *B. velezensis* [70] and *B. amyloliquefaciens* [71]. The antifungal capacity of these *Bacillus* strains was further evaluated for their potential application in tomato fruits. Therefore, an intermediate product-oriented approach was used based on TBA, mimicking the compositional and physicochemical properties of tomato, while avoiding the biological variability inherent in whole fruits (e.g., ripening stage). In addition, TBA reduces logistical challenges associated with large-scale fruit testing, while providing rapid and cost-effective preliminary data. The results on TBA showed, in general, low growth potential of the tested *Bacillus* isolates at pH 4.0, while at pH 6.0 some isolates showed moderate- or high-antifungal activity against *F. oxysporum* and *C. acutatum*. These results highlight the importance of testing the efficacy of potential candidates under actual food conditions to obtain optimal results for developing bio-preservation-based strategies. The ability of microorganisms, in general, to grow and maintain their active metabolism depends on each strain. Outside their optimal range, particularly in soil or rhizospheres, viability and population density may be reduced, affecting their ability to colonize roots and influencing their competition with other microorganisms for that ecological niche. Many species belonging to *Bacillus* spp. are known to tolerate a relatively wide pH range (5.0 to 8.0) thanks to its ability to form spores, which give them an advantage in acidic or alkaline conditions. Conversely, other microorganisms, such as *Pseudomonas fluorescens* or *Pseudomonas putida*, prefer neutral or slightly alkaline pH levels; in very acidic soils, their activity can decrease significantly. In our experience, we have tried to test an extreme context (pH = 4.0) and a context within the usual range of many *Bacillus* species (pH = 6.0). The results obtained demonstrated the remarkable antifungal activity of the strains described in Table 3. The results obtained in this work align with other recent studies that have also evaluated the antifungal activity of certain *Bacillus* species under acidic conditions. In this regard, Chowdhury et al. (2022) [72] demonstrated that the acid-tolerant *Bacillus amyloliquefaciens* MBNC efficiently produced antifungal lipopeptides and maintained a strong antagonistic activity against fungal pathogens under acidic conditions (pH 4.5). Notably, surfactin C15 was detected exclusively at pH 4.5, while surfactin homologs C12, C13, and C14 were present at both acidic and neutral pH. Under acidic conditions, *B. amyloliquefaciens* MBNC inhibited *Fusarium verticillioides* hyphal growth by 82%. Furthermore, the inhibitory effect was greater at pH 4.5 than at pH 7.0 against *F. oxysporum* and *Athelia rolfsii*. In another study, *B. velezensis* demonstrated significant antibacterial activity against *Ralstonia solanacearum* activity under acidic conditions (pH 4.0) [73]. In our work, among the nine *Bacillus* strains tested, only *B. aerius* BRZ3-2 was capable of growing at pH 4.0 following an adaptation period. Notably, *B. aerius* spores can germinate at pH 4.0, suggesting inherent acid-tolerance mechanisms that enable survival in low-pH environments, as reported by [74]. The unique ability of this strain to grow at pH 4.0 was associated with inhibition of *F. oxysporum* (25% inhibition) at low pH but not *C. acutatum*. These results are consistent with the fact that pH modulates the plant–microorganism relationship, as it affects bacterial survival and colonization, the potential production of bioactive compounds, and the expression of virulence [75] or beneficial genes. Therefore, the greater or lesser resistance to pH of each strain determines whether its effect on tomatoes (or other plants) will be stronger, weaker, or even null, depending on the environment [76].

At pH 6.0, seven isolates inhibited *F. oxysporum*, while ten suppressed *C. acutatum* growth, highlighting higher efficacy under near-optimal growth conditions. Such pH-dependent activity may stem from reduced stability of antifungal metabolites or enzymes under acidic conditions, as observed in studies where extreme pH levels reduce the antifungal ability of *Bacillus* species [77]. These results demonstrate that pH not only affects survival but also the expression of genes related to both protective and pathogenic functions, as in the case of the production of phytohormones and secondary metabolites. Thus, there are several studies that consider the ability of *Pseudomonas* spp. and *Bacillus* spp. plant growth promoters (PGPR) to produce auxins, siderophores, or substances with antimicrobial capacity, whose synthesis depends on pH [78,79]. In virtually all cases, pH 6.0 is ideal for this activity. Focusing on tomatoes, slightly acidic soils (pH 5.5–6.5) allow many strains of *Bacillus* to remain active and aid growth or protect against pathogens.

Further investigations of isolates TRB1-7 and BRZ3-2 are needed to evaluate their potential use as bioprotective cultures. These studies should include a comprehensive characterization of their antimicrobial capacity, including various mechanisms such as siderophore production [80], emission of volatile organic compounds [81], competition for nutrients and space, and biofilm formation [82]. Once these strains are fully characterized, it will be essential to assess their safety to ensure they are not harmful and eligible for GRAS (Generally Recognized as Safe) or QPS status. Moreover, studies should confirm their viability and functionality under storage and processing conditions, as well as their compliance with the technological requirements necessary to guarantee their effectiveness.

## 5. Conclusions

This study presents a stepwise approach to screen bacterial isolates with bioprotective potential from tomato plants. Results demonstrated that the higher performance in *Bacillus* spp. was in line with their abundance in the different anatomic parts and their better antimicrobial profiles. In contrast, while *Pseudomonas* spp. were also prevalent in tomato tissues, isolates exhibited relatively weak inhibitory activity against both bacterial and fungal targets. The combination of acid-adaptation protocols and use of an experimental model system helped evaluate the antimicrobial capacity under simulated tomato fruit conditions. Specifically, isolates TRB1-7 and BRZ3-2 showed promising inhibitory effects against *L. monocytogenes* and *F. oxysporum*, respectively, supporting their potential use as natural bioprotective agents. The findings underline the importance of exploring endogenous plant microbiota as sustainable alternatives to chemical preservatives and pesticides, contributing to food safety enhancement, waste reduction, and the development of eco-friendly preservation strategies within the agri-food sector. To enable practical bio-preservation applications, future work should include in vivo validation studies on tomato fruits of different cultivars and ripening stages under real post-harvest conditions, as well as genomic and metabolomic characterization of antimicrobial metabolites.

## Figures and Tables

**Table 1:** Percentages of *Bacillus* spp., *Pseudomonas* spp., and LAB isolated from each anatomical part of the tomato plant.

	Anatomical part			Total
	Fruit	Green Parts	Rhizosphere	
*Bacillus* spp.	7%	28%	8%	44%
*Pseudomonas* spp.	3%	42%	8%	52%
LAB	2%	2%	-	4%
Total	12%	72%	16%	100%

-: no bacteria were isolated.

**Table 2:** Antibacterial activity of *Bacillus* and *Pseudomonas* spp. isolates against *L. monocytogenes* and *P. carotovorum*.

Isolate Code	Genus	Isolation Origin	Target Bacteria	Halo Diameter (mm)	Inhibition
TRB1-1	*Bacillus*	Rhizosphere	*L. monocytogenes*	6.88 ± 1.01 *	Low
TRB1-2	*Bacillus*	Rhizosphere	*L. monocytogenes*	5.64 ± 1.40	Low
TRB1-7	*Bacillus*	Rhizosphere	*L. monocytogenes*	10.64 ± 0.78	Moderate
THP4-9	*Pseudomonas*	Leaves	*L. monocytogenes*	4.22 ± 0.76	Low
THB4-1	*Bacillus*	Leaves	*L. monocytogenes*	6.52 ± 0.85	Low
THB5-3	*Bacillus*	Leaves	*L. monocytogenes*	6.42 ± 0.45	Low
TTP4-1	*Pseudomonas*	Leaves	*L. monocytogenes*	8.63 ± 2.38	Low
TTP4-2	*Pseudomonas*	Leaves	*L. monocytogenes*	9.38 ± 1.10	Low
TTP4-6	*Pseudomonas*	Leaves	*P. carotovorum*	7.58 ± 1.42	Low
THB7-3	*Bacillus*	Leaves	*P. carotovorum*	8.27 ± 0.98	Low
THB7-9	*Bacillus*	Leaves	*P. carotovorum*	5.00 ± 1.03	Low
THB7-10	*Bacillus*	Leaves	*P. carotovorum*	6.93 ± 2.31	Low

* mean ± standard deviation.

**Table 3:** Phylogenetic similarity by 16S rRNA gene sequencing of selected bacterial strains isolated from tomato fruits and rhizospheres, and the percentage of inhibition (%) of *Bacillus* isolates against *F. oxysporum* and *C. acutatum* on PDA at pH 4.0 and pH 6.0.

Isolate Code	Isolation Origin	Antifungal Activity				E-Value	Identity	Name	Access Number
		*F. oxysporum*		*C. acutatum*					
		pH 4.0	pH 6.0	pH 4.0	pH 6.0				
TFB2-2	Fruit	**	38	**	41	0.0	100.00%	*Bacillus amyloliquefaciens*	ON722567.1
TFB3-1	Fruit	**	39	**	35	0.0	99.88%	*Bacillus* sp. *	MN508564.1
BT1-2	Fruit	**	40	**	35	0.0	100.00%	*Bacillus amyloliquefaciens*	CP113418.1
BT1-4	Fruit	**	**	**	33	0.0	100.00%	*Bacillus velezensis*	OQ244490.1
BT2-1	Fruit	**	39	**	32	0.0	99.86%	*Bacillus velezensis*	OQ244490.1
BRZ3-2	Rhizosphere	25	38	**	32	0.0	100.00%	*Bacillus aerius*	OM283596.1
TP1G	Fruit	**	38	**	24	0.0	99.89%	*Bacillus velezensis*	MN190156.1
TP2G	Fruit	**	34	**	33	0.0	100.00%	*Bacillus velezensis*	MN190156.1
TE2	Fruit	**	**	**	38	0.0	100.00%	*Bacillus amyloliquefaciens*	CP113418.1

* the sequencing did not reach species-level identification. ** no inhibition.

**Table 4:** Antifungal activity of three *Bacillus* isolates against *F. oxysporum* on TBA at pH 6.0, expressed as percentage of inhibition (%).

Isolate	Control Mold Diameter (mm)	Mold Diameter (mm)	Inhibition (%)
TFB3-1	6.56 ± 0.44 *	4.06 ± 0.17	38.11 ± 2.59
BT1-2	6.56 ± 0.44	4.30 ± 0.37	34.53 ± 5.71
BT1-4	6.56 ± 0.44	4.09 ± 0.13	37.73 ± 2.05

* mean ± standard deviation.

**Table 5:** Antifungal activity of ten *Bacillus* isolates against *C. acutatum* on TBA at pH 6.0, expressed as percentage of inhibition (%).

Isolate	Control Mold Diameter (mm)	Mold Diameter (mm)	Inhibition (%)
TFB3-1	2.25 ± 0.07 *	**	100
BT1-2	2.25 ± 0.07	**	100
BT1-4	2.25 ± 0.07	1.73 ± 0.15	23.11 ± 0.76
BT2-1	2.25 ± 0.07	**	100
BRZ3-2	2.25 ± 0.07	**	100
TP1G	2.25 ± 0.07	**	100
TP2G	2.25 ± 0.07	**	100
TE2	2.25 ± 0.07	**	100

* mean ± standard deviation. ** no fungal growth was observed.

## Data Availability

The original contributions presented in this study are included in the article and Supplementary Materials. Further inquiries can be directed to the corresponding author.

## Supplementary Materials

The following supporting information can be downloaded at: https://www.mdpi.com/article/10.3390/foods14213713/s1. Table S1. *Bacillus* spp., *Pseudomonas* spp. and lactic acid bacteria isolated from different anatomical parts (stems, leaves, fruit, and rhizosphere) of two tomato plants cultivars. Table S2. Antifungal activity of isolated bacteria against *F. oxysporum*, expressed as inhibition percentage (%). Table S3. Antifungal activity of isolated bacteria against *C. acutatum*, expressed as inhibition percentage (%). Figure S1. Examples of the two parameters followed to classify the antifungal activity. Figure S2. Positive antifungal activity results of the selected *Bacillus* spp. strains against *F. oxysporum* at pH 6.0. Figure S3. Positive antifungal activity results of the selected *Bacillus* spp. strains against *C. acutatum* at pH 6.0. Figure S4. Antifungal activity of the isolates TFB3-1, BT1-2, BT1-4, and BRZ3-2 at pH 4.0 and pH 6.0 in TBA against *F. oxysporum* and *C. acutatum* in 24-well plates. Figure S5. Antifungal activity of the *Bacillus* isolates TFB3-1, BT1-2, and BT1-4 against *F. oxysporum* at pH 6.0. Figure S6. Antifungal activity of the *Bacillus* isolates against *C. acutatum* at pH 6.0.

## Abbreviations

The following abbreviations are used in this manuscript:

EU	European Union
spp.	Species
HHP	High Hydrostatic Pressure
LAB	Lactic Acid Bacteria
OTUs	Operational Taxonomic Units
MRS	Man, Rogosa, and Sharpe
CFC	Cetrimide, Fucidin, and Cephalosporin
NA	Nutrient Agar
CECT	Spanish Type Culture Collection
PDA	Potato Dextrose Agar
PDB	Potato Dextrose Broth
TSB	Tryptone Soy Broth
TBA	Tomato-Based Agar
BHI	Brain Hearth Infusion
EFSA	European Food Safety Authority
QPS	Qualified Presumption of Safety
DNA	Deoxyribonucleic Acid
VOCs	Volatile Organic Compounds
ROS	Reactive Oxygen Species
GRAS	Generally Recognized As Safe
PGPR	Plant Growth Promoters
