# Supplementary material for: Screening of Microbial Isolates from Tomato Plants (Solanum lycopersicum L.) for Bioprotective Potential: From Isolation to Food Model System Application
Source: Foods. 2025 Oct 30;14(21):3713. doi: 10.3390/foods14213713 (PMC12607484; doi:10.3390/foods14213713)
Supplement: Supplementary file 1 [file foods-14-03713-s001.zip › foods-3917008-supplementary.pdf]

Table S1. *Bacillus* spp. *Pseudomonas* spp. and lactic acid bacteria isolated from different anatomical parts (stems, leaves, fruit and rhizosphere) of two tomato plants cultivar.

Anatomical part	Genera	Isolate code	Tomato cultivar
Stems	*Bacillus* spp.	TTB1-1	Harrison
Stems	*Bacillus* spp.	TTB1-2	Harrison
Stems	*Bacillus* spp.	TTB1-3	Harrison
Stems	*Bacillus* spp.	TTB2-1	Harrison
Stems	*Bacillus* spp.	TTB2-2	Harrison
Stems	*Bacillus* spp.	TTB3-1	Harrison
Stems	*Bacillus* spp.	TTB3-2	Harrison
Stems	*Pseudomonas* spp.	TTP1-1	Harrison
Stems	*Pseudomonas* spp.	TTP1-2	Harrison
Stems	*Pseudomonas* spp.	TTP2-1	Harrison
Stems	*Pseudomonas* spp.	TTP2-2	Harrison
Stems	*Pseudomonas* spp.	TTP1-3	Harrison
Stems	*Pseudomonas* spp.	TTP1-4	Harrison
Stems	*Pseudomonas* spp.	TTP2-3	Harrison
Stems	*Pseudomonas* spp.	TTP2-4	Harrison
Stems	*Pseudomonas* spp.	TTP1-5	Harrison
Stems	*Pseudomonas* spp.	TTP1-6	Harrison
Stems	*Pseudomonas* spp.	TTP1-7	Harrison
Stems	*Pseudomonas* spp.	TTP3-1	Harrison
Stems	*Pseudomonas* spp.	TTP3-2	Harrison
Stems	*Pseudomonas* spp.	TTP3-3	Harrison
Stems	*Bacillus* spp.	TTB3-1	Harrison
Stems	*Bacillus* spp.	TTB3-2	Harrison
Stems	*Bacillus* spp.	TTB3-3	Harrison
Stems	*Bacillus* spp.	TTB3-4	Harrison
Stems	*Bacillus* spp.	TTB4-1	Mistela
Stems	*Bacillus* spp.	TTB4-2	Mistela
Stems	*Bacillus* spp.	TTB4-3	Mistela
Stems	*Bacillus* spp.	TTB4-4	Mistela
Stems	*Bacillus* spp.	TTB4-5	Mistela
Stems	*Bacillus* spp.	TTB4-6	Mistela
Stems	*Bacillus* spp.	TTB4-7	Mistela
Stems	*Bacillus* spp.	TTB4-8	Mistela
Leaves	*Pseudomonas* spp.	THP1-1	Harrison
Leaves	*Pseudomonas* spp.	THP1-2	Harrison
Leaves	*Pseudomonas* spp.	THP1-3	Harrison
Leaves	*Pseudomonas* spp.	THP1-4	Harrison
Leaves	*Pseudomonas* spp.	THP2-3	Harrison
Leaves	*Pseudomonas* spp.	THP1-5	Harrison
Leaves	*Pseudomonas* spp.	THP2-4	Harrison
Leaves	*Pseudomonas* spp.	THP2-1	Harrison
Leaves	*Pseudomonas* spp.	THP2-2	Harrison
Leaves	*Bacillus* spp.	THB1-1	Harrison

Leaves	*Bacillus* spp.	THB2-1	Harrison
Leaves	*Bacillus* spp.	THB2-2	Harrison
Leaves	*Bacillus* spp.	THB1-2	Harrison
Leaves	*Bacillus* spp.	THB1-3	Harrison
Leaves	*Bacillus* spp.	THB1-4	Harrison
Leaves	*Bacillus* spp.	THB2-3	Harrison
Leaves	BAL	THA1-1	Harrison
Leaves	BAL	THA1-2	Harrison
Leaves	BAL	THA1-3	Harrison
Leaves	*Pseudomonas* spp.	THP3-1	Harrison
Leaves	*Pseudomonas* spp.	THP3-2	Harrison
Leaves	*Pseudomonas* spp.	THP3-3	Harrison
Leaves	*Pseudomonas* spp.	THP3-4	Harrison
Leaves	*Pseudomonas* spp.	THP3-5	Harrison
Leaves	*Pseudomonas* spp.	THP3-6	Harrison
Leaves	*Pseudomonas* spp.	THP3-7	Harrison
Leaves	*Pseudomonas* spp.	THP3-8	Harrison
Leaves	*Pseudomonas* spp.	THP3-9	Harrison
Leaves	*Pseudomonas* spp.	THP3-10	Harrison
Leaves	*Pseudomonas* spp.	THP3-11	Harrison
Leaves	*Pseudomonas* spp.	THP3-12	Harrison
Leaves	*Pseudomonas* spp.	THP3-13	Harrison
Leaves	*Pseudomonas* spp.	THP3-14	Harrison
Leaves	*Pseudomonas* spp.	THP3-15	Harrison
Leaves	*Bacillus* spp.	THB3-1	Harrison
Leaves	*Bacillus* spp.	THB3-2	Harrison
Leaves	*Bacillus* spp.	THB3-3	Harrison
Leaves	*Bacillus* spp.	THB3-4	Harrison
Leaves	*Bacillus* spp.	THB3-5	Harrison
Leaves	*Bacillus* spp.	THB3-6	Harrison
Leaves	*Pseudomonas* spp.	THP4-1	Harrison
Leaves	*Pseudomonas* spp.	THP4-2	Harrison
Leaves	*Pseudomonas* spp.	THP4-3	Harrison
Leaves	*Pseudomonas* spp.	THP4-4	Harrison
Leaves	*Pseudomonas* spp.	THP4-5	Harrison
Leaves	*Pseudomonas* spp.	THP4-6	Harrison
Leaves	*Pseudomonas* spp.	THP4-7	Harrison
Leaves	*Pseudomonas* spp.	THP4-8	Harrison
Leaves	*Pseudomonas* spp.	THP4-9	Harrison
Leaves	*Pseudomonas* spp.	THP4-10	Harrison
Leaves	*Pseudomonas* spp.	THP4-11	Harrison
Leaves	*Pseudomonas* spp.	THP4-12	Harrison
Leaves	*Pseudomonas* spp.	THP5-1	Harrison
Leaves	*Pseudomonas* spp.	THP5-2	Harrison
Leaves	*Pseudomonas* spp.	THP5-3	Harrison
Leaves	*Pseudomonas* spp.	THP5-4	Harrison
Leaves	*Pseudomonas* spp.	THP5-5	Harrison

Leaves	*Pseudomonas* spp.	THP5-6	Harrison
Leaves	*Pseudomonas* spp.	THP5-7	Harrison
Leaves	*Pseudomonas* spp.	THP5-8	Harrison
Leaves	*Pseudomonas* spp.	THP5-9	Harrison
Leaves	*Pseudomonas* spp.	THP5-10	Harrison
Leaves	*Pseudomonas* spp.	THP5-11	Harrison
Leaves	*Pseudomonas* spp.	THP5-12	Harrison
Leaves	*Pseudomonas* spp.	THP5-13	Harrison
Leaves	*Pseudomonas* spp.	THP5-14	Harrison
Leaves	*Pseudomonas* spp.	THP6-1	Harrison
Leaves	*Pseudomonas* spp.	THP6-2	Harrison
Leaves	*Pseudomonas* spp.	THP6-3	Harrison
Leaves	*Pseudomonas* spp.	THP6-4	Harrison
Leaves	*Pseudomonas* spp.	THP6-5	Harrison
Leaves	*Pseudomonas* spp.	THP6-6	Harrison
Leaves	*Pseudomonas* spp.	THP6-7	Harrison
Leaves	*Pseudomonas* spp.	THP6-8	Harrison
Leaves	*Pseudomonas* spp.	THP6-9	Harrison
Leaves	*Pseudomonas* spp.	THP6-10	Harrison
Leaves	*Pseudomonas* spp.	THP6-11	Harrison
Leaves	*Pseudomonas* spp.	THP6-12	Harrison
Leaves	*Pseudomonas* spp.	THP7-1	Harrison
Leaves	*Pseudomonas* spp.	THP7-2	Harrison
Leaves	*Pseudomonas* spp.	THP7-3	Harrison
Leaves	*Pseudomonas* spp.	THP7-4	Harrison
Leaves	*Pseudomonas* spp.	THP7-5	Harrison
Leaves	*Pseudomonas* spp.	THP7-6	Harrison
Leaves	*Pseudomonas* spp.	THP7-7	Harrison
Leaves	*Pseudomonas* spp.	THP7-8	Harrison
Leaves	*Pseudomonas* spp.	THP7-9	Harrison
Leaves	*Pseudomonas* spp.	THP7-10	Harrison
Leaves	*Pseudomonas* spp.	THP7-11	Harrison
Leaves	*Pseudomonas* spp.	THP7-12	Harrison
Leaves	*Bacillus* spp.	THB4-1	Harrison
Leaves	*Bacillus* spp.	THB4-2	Harrison
Leaves	*Bacillus* spp.	THB4-3	Harrison
Leaves	*Bacillus* spp.	THB4-4	Harrison
Leaves	*Bacillus* spp.	THB4-5	Harrison
Leaves	*Bacillus* spp.	THB4-6	Harrison
Leaves	*Bacillus* spp.	THB4-7	Harrison
Leaves	*Bacillus* spp.	THB4-8	Harrison
Leaves	*Bacillus* spp.	THB5-1	Harrison
Leaves	*Bacillus* spp.	THB5-2	Harrison
Leaves	*Bacillus* spp.	THB5-3	Harrison
Leaves	*Bacillus* spp.	THB5-4	Harrison
Leaves	*Bacillus* spp.	THB5-5	Harrison
Leaves	*Bacillus* spp.	THB5-6	Harrison

Leaves	*Bacillus* spp.	THB5-7	Harrison
Leaves	*Bacillus* spp.	THB5-8	Harrison
Leaves	*Bacillus* spp.	THB6-1	Harrison
Leaves	*Bacillus* spp.	THB6-2	Harrison
Leaves	*Bacillus* spp.	THB6-3	Harrison
Leaves	*Bacillus* spp.	THB6-4	Harrison
Leaves	*Bacillus* spp.	THB6-5	Harrison
Leaves	*Bacillus* spp.	THB6-6	Harrison
Leaves	*Bacillus* spp.	THB6-7	Harrison
Leaves	*Bacillus* spp.	THB6-8	Harrison
Leaves	*Pseudomonas* spp.	TTP4-1	Mistela
Leaves	*Pseudomonas* spp.	TTP4-2	Mistela
Leaves	*Pseudomonas* spp.	TTP4-3	Mistela
Leaves	*Pseudomonas* spp.	TTP4-4	Mistela
Leaves	*Pseudomonas* spp.	TTP4-5	Mistela
Leaves	*Pseudomonas* spp.	TTP4-6	Mistela
Leaves	*Pseudomonas* spp.	TTP4-7	Mistela
Leaves	*Pseudomonas* spp.	TTP4-8	Mistela
Leaves	*Pseudomonas* spp.	THP8-1	Mistela
Leaves	*Pseudomonas* spp.	THP8-2	Mistela
Leaves	*Pseudomonas* spp.	THP8-3	Mistela
Leaves	*Pseudomonas* spp.	THP8-4	Mistela
Leaves	*Pseudomonas* spp.	THP8-5	Mistela
Leaves	*Pseudomonas* spp.	THP8-6	Mistela
Leaves	*Pseudomonas* spp.	THP8-7	Mistela
Leaves	*Pseudomonas* spp.	THP8-8	Mistela
Leaves	*Bacillus* spp.	THB7-1	Mistela
Leaves	*Bacillus* spp.	THB7-2	Mistela
Leaves	*Bacillus* spp.	THB7-3	Mistela
Leaves	*Bacillus* spp.	THB7-4	Mistela
Leaves	*Bacillus* spp.	THB7-5	Mistela
Leaves	*Bacillus* spp.	THB7-6	Mistela
Leaves	*Bacillus* spp.	THB7-7	Mistela
Leaves	*Bacillus* spp.	THB7-8	Mistela
Leaves	*Bacillus* spp.	THB7-9	Mistela
Leaves	*Bacillus* spp.	THB7-10	Mistela
Leaves	*Bacillus* spp.	THB7-11	Mistela
Leaves	*Bacillus* spp.	THB7-12	Mistela
Leaves	*Bacillus* spp.	THB7-13	Mistela
Leaves	*Bacillus* spp.	THB7-14	Mistela
Leaves	LAB	THA2-1	Mistela
Leaves	LAB	THA2-2	Mistela
Fruit	*Pseudomonas* spp.	TFP1-1	Harrison
Fruit	*Bacillus* spp.	TFB1-1	Harrison
Fruit	*Bacillus* spp.	TFB1-2	Harrison
Fruit	*Bacillus* spp.	TFB1-3	Harrison
Fruit	*Bacillus* spp.	TFB1-4	Harrison

Fruit	*Bacillus* spp.	TFB2-1	Harrison
Fruit	*Bacillus* spp.	TFB2-2	Harrison
Fruit	*Bacillus* spp.	TFB3-1	Harrison
Fruit	*Bacillus* spp.	TFB3-2	Harrison
Fruit	*Bacillus* spp.	TFB3-3	Harrison
Fruit	*Pseudomonas* spp.	TFP2-1	Mistela
Fruit	*Pseudomonas* spp.	TFP2-2	Mistela
Fruit	*Pseudomonas* spp.	PT-1 1	Mistela
Fruit	*Pseudomonas* spp.	PT-1 2	Mistela
Fruit	*Pseudomonas* spp.	PT-1 3	Mistela
Fruit	*Pseudomonas* spp.	PT-1 4	Mistela
Fruit	*Bacillus* spp.	BT-1 2	Mistela
Fruit	*Bacillus* spp.	BT-1 3	Mistela
Fruit	*Bacillus* spp.	BT-2 1	Mistela
Fruit	*Bacillus* spp.	BT-2 2	Mistela
Fruit	*Bacillus* spp.	BT-1 4	Mistela
Fruit	LAB	BT 1	Mistela
Fruit	LAB	BT 2	Mistela
Fruit	LAB	BT 3	Mistela
Fruit	LAB	BT 4	Mistela
Fruit	LAB	BT 5	Mistela
Fruit	*Bacillus* spp.	TP1G	Harrison
Fruit	*Bacillus* spp.	TP2G	Harrison
Fruit	*Bacillus* spp.	TE2	Harrison
Rhizosphere	*Pseudomonas* spp.	TRP1-1	Harrison
Rhizosphere	*Pseudomonas* spp.	TRP1-2	Harrison
Rhizosphere	*Pseudomonas* spp.	TRP1-3	Harrison
Rhizosphere	*Pseudomonas* spp.	TRP1-4	Harrison
Rhizosphere	*Pseudomonas* spp.	TRP1-5	Harrison
Rhizosphere	*Pseudomonas* spp.	TRP1-6	Harrison
Rhizosphere	*Pseudomonas* spp.	TRP1-7	Harrison
Rhizosphere	*Pseudomonas* spp.	TRP1-8	Harrison
Rhizosphere	*Pseudomonas* spp.	TRP1-9	Harrison
Rhizosphere	*Pseudomonas* spp.	TRP2-1	Harrison
Rhizosphere	*Pseudomonas* spp.	TRP2-2	Harrison
Rhizosphere	*Pseudomonas* spp.	TRP2-3	Harrison
Rhizosphere	*Bacillus* spp.	TRB1-1	Harrison
Rhizosphere	*Bacillus* spp.	TRB1-2	Harrison
Rhizosphere	*Bacillus* spp.	TRB1-3	Harrison
Rhizosphere	*Bacillus* spp.	TRB1-4	Harrison
Rhizosphere	*Bacillus* spp.	TRB1-5	Harrison
Rhizosphere	*Bacillus* spp.	TRB1-6	Harrison
Rhizosphere	*Bacillus* spp.	TRB1-7	Harrison
Rhizosphere	*Bacillus* spp.	TRB1-8	Harrison
Rhizosphere	*Bacillus* spp.	TRB1-9	Harrison
Rhizosphere	*Bacillus* spp.	TRB1-10	Harrison
Rhizosphere	*Bacillus* spp.	TRB1-11	Harrison

Rhizosphere	*Bacillus* spp.	TRB1-12	Harrison
Rhizosphere	*Bacillus* spp.	TRB1-13	Harrison
Rhizosphere	*Bacillus* spp.	TRB1-14	Harrison
Rhizosphere	*Bacillus* spp.	TRB1-15	Harrison
Rhizosphere	*Pseudomonas* spp.	PRZ-4 1	Mistela
Rhizosphere	*Pseudomonas* spp.	PRZ-4 2	Mistela
Rhizosphere	*Pseudomonas* spp.	PRZ-4 3	Mistela
Rhizosphere	*Pseudomonas* spp.	PRZ-5 1	Mistela
Rhizosphere	*Pseudomonas* spp.	PRZ-5 2	Mistela
Rhizosphere	*Pseudomonas* spp.	PRZ-5 3	Mistela
Rhizosphere	*Bacillus* spp.	BRZ-3 1	Mistela
Rhizosphere	*Bacillus* spp.	BRZ-3 2	Mistela
Rhizosphere	*Bacillus* spp.	BRZ-4 3	Mistela
Rhizosphere	*Bacillus* spp.	BRZ-5 1	Mistela

Table S2. Antifungal activity of isolated bacteria against *F. oxysporum*, expressed as inhibition percentage (%).

Isolate code	Genera	Anatomical part	Inhibition (%)	Inhibition (distance, mm)
THP5-8	*Pseudomonas* spp.	Leaves	18.94	NA
THB4-1	*Bacillus* spp.	Leaves	26.23	NA
THB4-2	*Bacillus* spp.	Leaves	24.67	NA
THB4-3	*Bacillus* spp.	Leaves	24.45	NA
THB4-6	*Bacillus* spp.	Leaves	19.68	NA
THB4-7	*Bacillus* spp.	Leaves	26.16	NA
THB5-2	*Bacillus* spp.	Leaves	28.30	NA
THB5-3	*Bacillus* spp.	Leaves	27.42	NA
THB5-4	*Bacillus* spp.	Leaves	26.12	NA
THB6-3	*Bacillus* spp.	Leaves	23.53	NA
TFB1-2	*Bacillus* spp.	Fruit	20.93	NA
TFB2-2	*Bacillus* spp.	Fruit	21.88	NA
THP8-5	*Pseudomonas* spp.	Leaves	10.89	NA
THP8-6	*Pseudomonas* spp.	Leaves	23.62	NA
THP8-7	*Pseudomonas* spp.	Leaves	18.97	NA
THP8-8	*Pseudomonas* spp.	Leaves	17.88	NA
THB7-1	*Bacillus* spp.	Leaves	24.56	NA
THB7-2	*Bacillus* spp.	Leaves	17.98	NA
THB7-3	*Bacillus* spp.	Leaves	20.66	NA
THB7-4	*Bacillus* spp.	Leaves	24.53	NA
THB7-6	*Bacillus* spp.	Leaves	36.07	NA
THB7-7	*Bacillus* spp.	Leaves	26.07	NA
THB7-9	*Bacillus* spp.	Leaves	33.00	NA
THB7-11	*Bacillus* spp.	Leaves	28.55	NA

THB7-12	*Bacillus* spp.	Leaves	28.21	NA
TTB4-1	*Bacillus* spp.	Stems	27.74	NA
TTB4-2	*Bacillus* spp.	Stems	22.86	NA
TTB4-5	*Bacillus* spp.	Stems	25.48	NA
TTB4-6	*Bacillus* spp.	Stems	30.38	NA
THA2-1	LAB	Leaves	17.17	NA
THA2-2	LAB	Leaves	16.26	NA
BRZ3-2	*Bacillus* spp.	Fruit	37.63	NA
PRZ-4 1	*Pseudomonas* spp.	Rhizosphere	19.49	NA
TFB2-2	*Bacillus* spp.	Fruit	38.46	NA
TFB3-1	*Bacillus* spp.	Fruit	38.63	NA
BT1-2	*Bacillus* spp.	Fruit	39.63	NA
BT2-1	*Bacillus* spp.	Fruit	38.80	NA
TP1G	*Bacillus* spp.	Fruit	37.59	NA
TP2G	*Bacillus* spp.	Fruit	33.95	NA

NA: Not Applicable

Table S3. Antifungal activity of isolated bacteria against *C. acutatum*, expressed as inhibition percentage (%).

Isolate code	Genera	Anatomical part	Inhibition (%)	Inhibition (distance, mm)
THP4-11	*Pseudomonas* spp.	Leaves	21.97	NA
THP4-12	*Pseudomonas* spp.	Leaves	22.93	NA
THP5-5	*Pseudomonas* spp.	Leaves	22.05	NA
THB4-2	*Bacillus* spp.	Leaves	48.04	NA
THB4-3	*Bacillus* spp.	Leaves	47.82	NA
THB4-6	*Bacillus* spp.	Leaves	41.82	NA
THB4-7	*Bacillus* spp.	Leaves	56.42	NA
THB5-2	*Bacillus* spp.	Leaves	49.70	NA
THB5-3	*Bacillus* spp.	Leaves	75.57	NA
THB5-4	*Bacillus* spp.	Leaves	61.37	NA
THB6-3	*Bacillus* spp.	Leaves	73.94	NA
TFB2-2	*Bacillus* spp.	Fruit	40.91	NA
TFB3-1	*Bacillus* spp.	Fruit	35.28	NA
TTP4-6	*Pseudomonas* spp.	Stems	32.63	NA
THP8-6	*Pseudomonas* spp.	Leaves	48.77	NA
THP8-8	*Pseudomonas* spp.	Leaves	33.88	NA
THB7-1	*Bacillus* spp.	Leaves	59.47	NA
THB7-2	*Bacillus* spp.	Leaves	31.41	NA
THB7-3	*Bacillus* spp.	Leaves	38.53	NA
THB7-4	*Bacillus* spp.	Leaves	45.25	NA
THB7-6	*Bacillus* spp.	Leaves	63.89	NA
THB7-7	*Bacillus* spp.	Leaves	50.40	NA
THB7-11	*Bacillus* spp.	Leaves	45.31	NA
THB7-12	*Bacillus* spp.	Leaves	68.04	NA
THB7-13	*Bacillus* spp.	Leaves	41.70	NA

TTB4-1	*Bacillus* spp.	Stems	74.52	NA
TTB4-5	*Bacillus* spp.	Stems	46.62	NA
TTB4-6	*Bacillus* spp.	Stems	54.04	NA
TTB4-7	*Bacillus* spp.	Stems	41.85	NA
THA2-1	LAB	Leaves	34.69	NA
THA2-2	LAB	Leaves	58.75	NA
BRZ3-2	*Bacillus* spp.	Rhizosphere	32.00	NA
BT1-2	*Bacillus* spp.	Fruit	34.85	NA
BT2-1	*Bacillus* spp.	Fruit	32.03	NA
BT1-4	*Bacillus* spp.	Fruit	32,25	NA
TP1G	*Bacillus* spp.	Fruit	34.42	NA
TP2G	*Bacillus* spp.	Fruit	33.33	NA
TE2	*Bacillus* spp.	Fruit	37.88	NA

NA: Not Applicable

**Parameter 1**<br><br>Distance between the mold and the bacterium drops (mm)	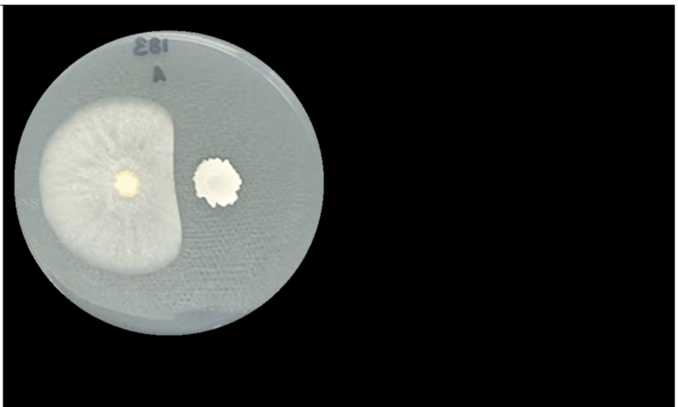
**Parameter 2**<br><br>Reduction of the mold diameter (a) in comparison to the control (b)	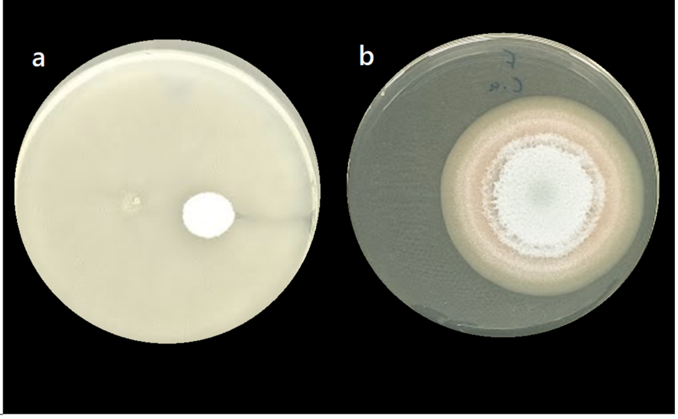

Figure S1. Examples of the two parameters followed to classify the antifungal activity

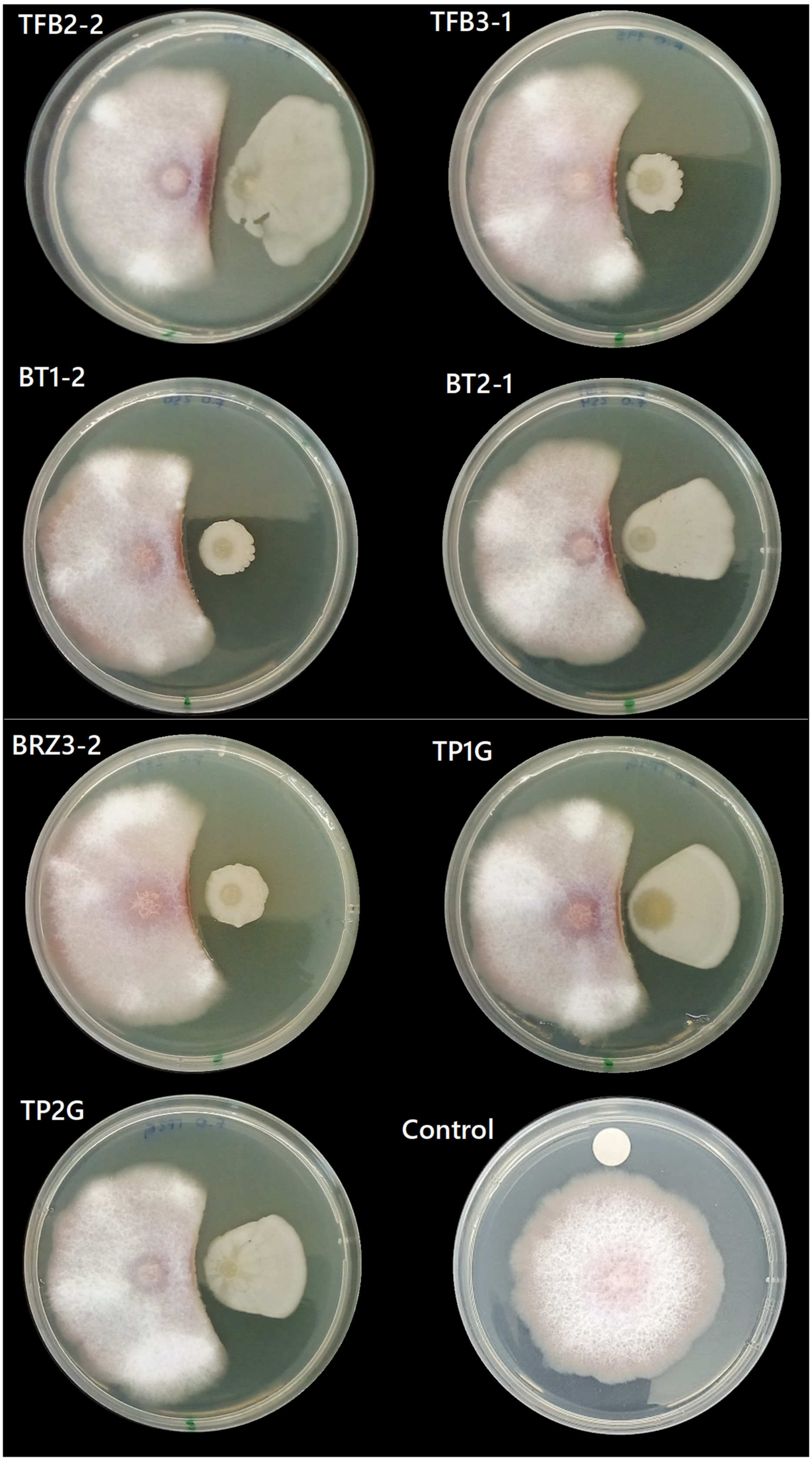

Figure S2. Positive antifungal activity results of the selected *Bacillus* spp. strains against *F. oxysporum* at pH 6.0

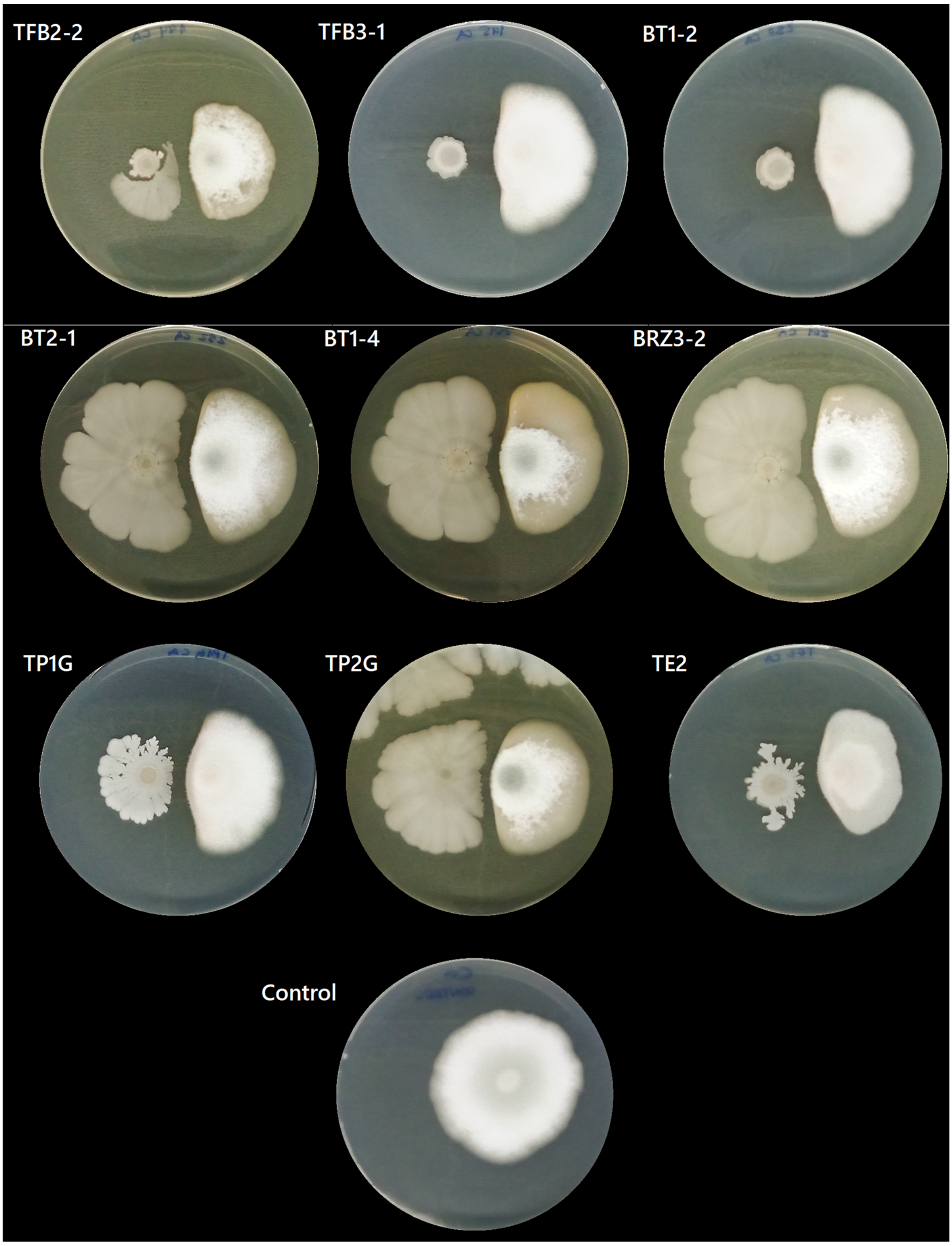

Figure S3. Positive antifungal activity results of the selected *Bacillus* spp. strains against *C. acutatum* at pH 6.0

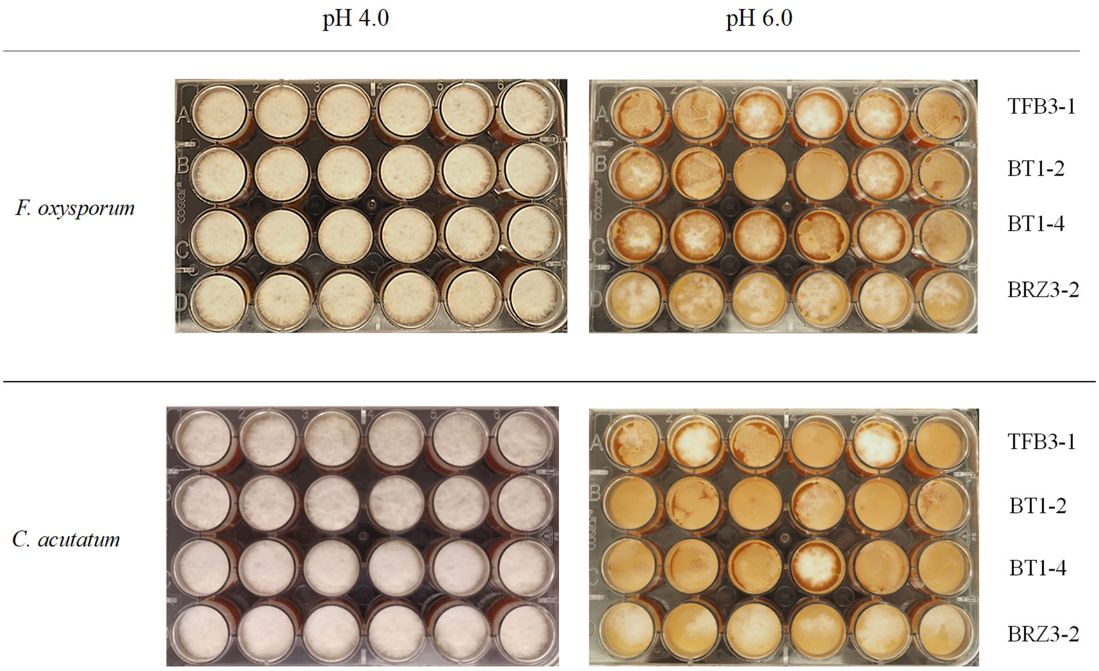

Figure S4. Antifungal activity of the isolates TFB3-1, BT1-2, BT1-4 and BRZ3-2 at pH 4.0 and pH 6.0 in TBA against *F. oxysporum* and *C. acutatum* in 24-well plates.

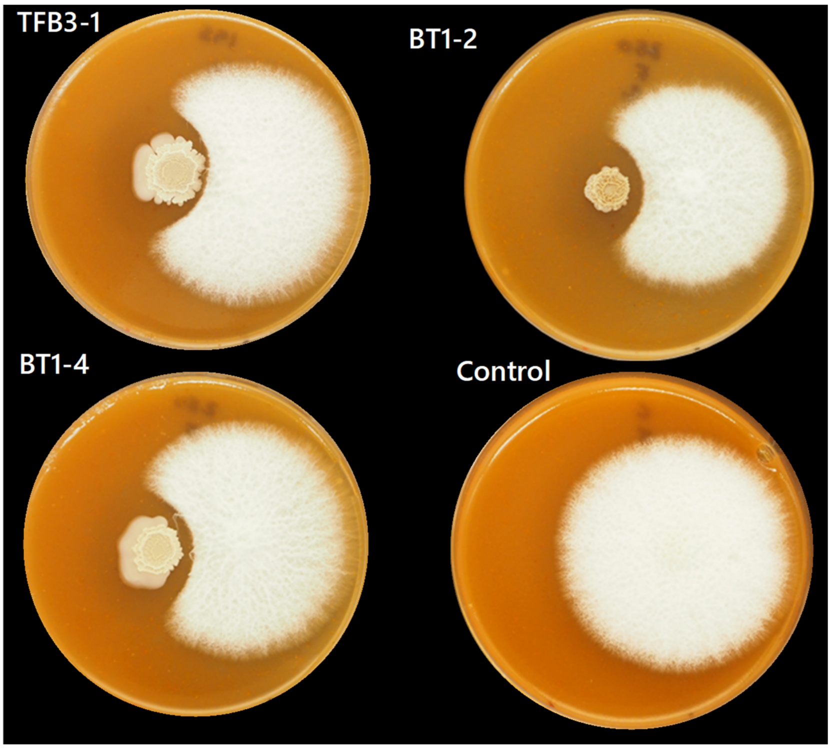

Figure S5. Antifungal activity of the *Bacillus* isolates TFB3-1, BT1-2 and BT1-4 against *F. oxysporum* at pH 6.0

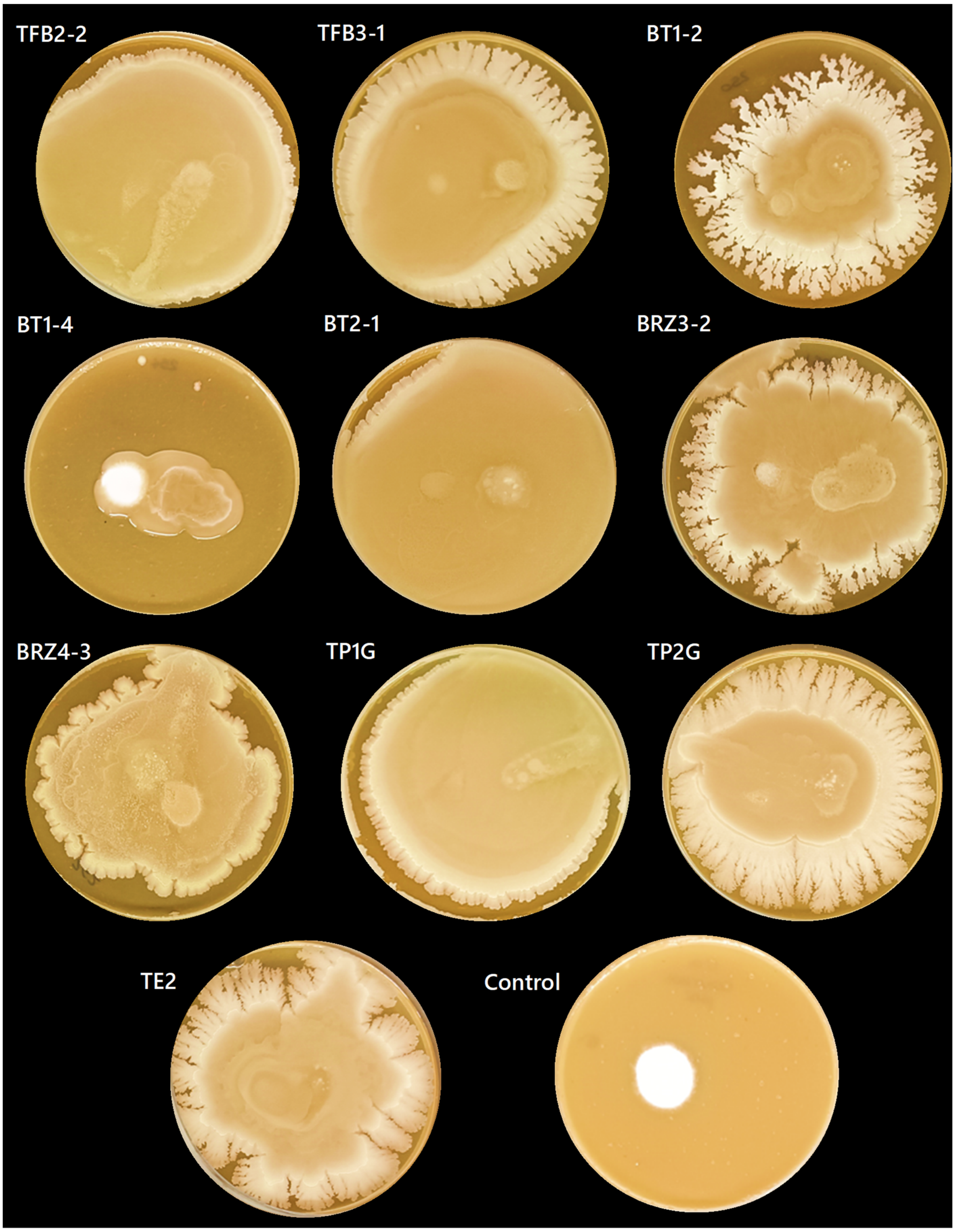

Figure S6. Antifungal activity of the *Bacillus* isolates against *C. acutatum* at pH 6.0
